# Amiodarone exacerbates brain injuries after hypoxic–ischemic insult in mice

**DOI:** 10.1186/s12868-019-0544-2

**Published:** 2019-12-21

**Authors:** Masakazu Kotoda, Sohei Hishiyama, Tadahiko Ishiyama, Kazuha Mitsui, Takashi Matsukawa

**Affiliations:** 1 FM Kirby Neurobiology Center, Boston Children’s Hospital, Harvard Medical School, Boston, USA; 2 Department of Anesthesiology, Faculty of Medicine, University of Yamanashi, 1110 Shimokato, Chuo, Yamanashi 409-3898 Japan; 3 Surgical Center, University of Yamanashi Hospital, University of Yamanashi, 1110 Shimokato, Chuo, Yamanashi 409-3898 Japan

**Keywords:** Amiodarone, Hypoxic–ischemic brain injury, Sodium ion

## Abstract

**Background:**

Sodium ion transportation plays a crucial role in the pathogenesis of hypoxic–ischemic brain injury. Amiodarone, a Vaughan-Williams class III antiarrhythmic drug, has been widely used to treat life-threatening arrhythmia and cardiac arrest worldwide. In addition to its inhibitory effects on the potassium channel, amiodarone also blocks various sodium ion transporters, including the voltage-gated sodium channel, sodium pump, and Na^+^/Ca^+^ exchanger. Considering these pharmacological profile, amiodarone may affect the influx–efflux balance of sodium ion in the hypoxic–ischemic brain. Previous studies suggest that the blockade of the voltage-gated sodium channel during hypoxic–ischemic brain injury exerts neuroprotection. On the contrary, the blockade of sodium pump or Na^+^/Ca^+^ exchanger during hypoxia–ischemia may cause further intracellular sodium accumulation and consequent osmotic cell death. From these perspectives, the effects of amiodarone on sodium ion balance on the hypoxic–ischemic brain can be both protective and detrimental depending on the clinical and pathophysiological conditions. In this study, we therefore investigated the effect of amiodarone on hypoxic–ischemic brain injury using a murine experimental model.

**Results:**

Compared with the control group mice, mice that received amiodarone after induction of 40-min hypoxic–ischemic brain injury exhibited lower survival rates over 7 days and worse neurological function. After 25-min hypoxic–ischemic brain injury, amiodarone treated mice exhibited larger infarct volumes (16.0 ± 6.9 vs. 24.2 ± 6.8 mm^3^, P < 0.05) and worse neurological function. In addition, the brains harvested from the amiodarone-treated mice contained larger amounts of sodium (194.7 ± 45.1 vs. 253.5 ± 50.9 mEq/kg dry weight, P < 0.01) and water (259.3 ± 8.9 vs. 277.2 ± 12.5 mg, P < 0.01). There were no significant differences in hemodynamic parameters between groups.

**Conclusions:**

Amiodarone exacerbated brain injuries and neurological outcomes after hypoxic–ischemic insults. Severe brain sodium accumulation and brain edema were associated with the detrimental effects of amiodarone. Amiodarone at the clinical dose can exacerbate brain injury after hypoxic–ischemic insult by affecting sodium ion transportation and facilitate intracellular sodium accumulation in the brain.

## Background

Sodium ion transportation plays a crucial role in the pathogenesis of hypoxic–ischemic brain injury [1, 2]. After hypoxic–ischemic insult, depletion of oxygen and cellular ATP leads to inactivation of the sodium pump (Na^+^–K^+^–ATPase). As a result, intracellular sodium accumulates, and the brain cell membrane becomes depolarized. Subsequently, activation of voltage-gated sodium channels occurs, which facilitates further accumulation of intracellular sodium, resulting in osmotic swelling and consequent necrotic cell death [1, 2].

Amiodarone, a Vaughan-Williams class III antiarrhythmic drug, has been widely used to treat life-threatening arrhythmia and cardiac arrest worldwide [3]. Although it is well known that amiodarone indirectly contributes to prevent ischemic stroke by treating atrial fibrillation [4], the direct effect of amiodarone on hypoxic–ischemic brain injury remains poorly understood. In addition to its inhibitory effect on potassium channels, amiodarone also blocks various sodium ion transporters, including the voltage-gated sodium channel, sodium pump, and Na^+^/Ca^+^ exchanger [4, 5]. Considering these pharmacological profile, amiodarone may affect the influx–efflux balance of sodium ion in the brain during hypoxic–ischemic insult. Previous studies suggest that the blockade of voltage-gated sodium channel during hypoxic–ischemic brain injury exerts neuroprotection via suppression of sodium ion influx and cellular hyper-excitability [2, 6]. On the contrary, the blockade of sodium pump or Na^+^/Ca^+^ exchangers during hypoxia–ischemia may cause further intracellular sodium accumulation, brain edema, and consequent osmotic cell death [1, 7, 8]. From these perspectives, the effects of amiodarone on sodium ion balance on the hypoxic–ischemic brain can be both protective and detrimental depending on clinical and pathophysiological conditions. Amiodarone is currently a second-line drug for the treatment of ventricular fibrillation during cardiopulmonary resuscitation. Cardiopulmonary arrest leads to brain hypoxia–ischemia, and the effect of amiodarone on the neurological prognosis and functional recovery after resuscitation is of great concern.

In the present study, we therefore investigated the effect of amiodarone at the clinical dose on hypoxic–ischemic brain injury using a murine experimental model. The primary outcomes of this study were survival rate and neurological function after hypoxic–ischemic brain injury. The secondary outcomes were infarct volumes, brain sodium content, and brain edema.

## Methods

### Animals

Male C57BL/6 mice (8–10-week old, weigh: 20–25 g) were purchased from Japan SLC (Tokyo, Japan). The mice were housed at 23 ± 2 °C under a 12-h light–dark cycle with free access to standard food and water. All experiments were performed between 09:00 and 17:00 under normal room light and temperature (23 ± 2 °C) conditions. A total of 44 mice were used in this study.

### Hypoxic–ischemic brain injury

Hypoxic–ischemic brain injury was induced via a combination of permanent left common carotid artery occlusion and exposure to a low-oxygen environment, as previously described [9, 10]. Briefly, mice were placed in a dorsal position, and a middle neck incision was made under isoflurane anesthesia. The left common carotid artery was isolated from the vagus nerve, and then ligated and cut. One hour after the common carotid artery occlusion, mice were exposed to a low-oxygen environment (8% O_2_ balanced with nitrogen) for either 40 min or 25 min. In the 40-min injury model, the amiodarone groups received a single-bolus intraperitoneal injection of amiodarone (Sanofi K.K., Tokyo, Japan, 50 mg/kg) either immediately (0 min) or 10 min after the induction of hypoxic–ischemic brain injury (n = 10 and 6 respectively), while the control group received only normal saline immediately after the induction hypoxic–ischemic brain injury (n = 10). In the 25-min injury model, the amiodarone group received 50 mg/kg amiodarone immediately after the induction of hypoxic–ischemic brain injury, while the control group received only normal saline. Two mice were excluded due to the death before 24 h after induction (one per each group); eight mice were included in each group. The amiodarone dose was chosen based on the body surface area [11] and previously published data [12]. The rectal temperature was monitored and maintained at 37 ± 0.5 °C using a heating pad. Heart rates and non-invasive blood pressures were monitored during the hypoxic–ischemic brain injury.

In the 40-min hypoxic–ischemic injury model, overall neurological function and survival rates over 7 days were evaluated. The neurological deficit score was used to evaluate global neurological deficit as follows: 0—no deficit; 1—torso flexion; 2—spontaneous circling; 3—leaning or longitudinal circling; 4—no spontaneous movement; 5—death [13]. In the 25-min injury model, the mice were euthanized 24 h after the hypoxia–ischemic insult, followed by infarct volume analysis or water/sodium content analysis.

### Measurement of infarct volume

Twenty-four hours after the 25-min hypoxic–ischemic injury, mice were deeply anesthetized with 5% isoflurane and euthanized by cervical dislocation. Brains were removed and coronal slices with a thickness of 1 mm were prepared. Brain slices were immersed in 2% 2,3,5-triphenyltetrazolium chloride (TTC; Sigma Aldrich, St. Louis, MO) solution, and incubated at 37 °C for 15 min. The area of infarction was traced and measured using image analysis software (ImageJ; National Institutes of Health, Bethesda, MD). The infarct area was calculated as follows to correct for edema: [1 − (total ipsilateral hemisphere − infarct region)/total contralateral hemisphere] × 100% [14]. Total infarct volume was calculated as the sum of all infarct areas multiplied by section thickness (n = 8 each).

### Evaluation of brain water content

Twenty-four hours after the 25-min hypoxic–ischemic injury, mice were deeply anesthetized with 5% isoflurane and euthanized by cervical dislocation. The whole brain was harvested and the olfactory bulbs and cerebellum were removed. The wet-weight of the brain was measured using a digital scale, and the brain was then freeze-dried for 72 h. The difference between the wet-weight and dry-weight was defined as brain water content (n = 8 each).

### Inductively coupled plasma analysis

The freeze-dried brain was subjected to mortar pulverization, and immersed in 13 M nitric acid on a hot plate for chemical decomposition. The solution was then diluted with distilled water into pH 3–4, and subjected to inductively coupled plasma analysis (SPS3520, Hitachi High-Technologies, Tokyo, Japan). Sodium concentration of the sample solutions and the total brain sodium content was determined via the method of standard addition. Values were expressed in mEq/kg dry weight (n = 8 each).

### Hemodynamic measurements

Three-needle probe electrocardiogram monitoring was performed during general anesthesia and the surgical procedures (Powerlab, Bioamp, and LabChart 8, AD Instruments, NSW, Australia). The arterial blood pressure was non-invasively measured using a tail-cuff method (Softron, Tokyo, Japan). In the 25-min hypoxic–ischemic injury model, these hemodynamic parameters were recorded immediately before induction of hypoxic–ischemic injury (baseline) and immediately after exposure to the hypoxic environment (n = 8 each).

### Statistical analysis

Statistical analysis was performed using Prism 6 software (GraphPad Software, San Diego, CA). The Log-rank test was used to analyze the survival rates; a two-tailed t test was used for the infarct volume, water content, and sodium content analysis; and the Kruskal–Wallis test followed by Dunn’s post hoc test was used to analyze neurological deficit scores. Values are presented as mean ± standard deviation in the infarct volume, water content, and sodium content analysis; values are presented as median and range in the neurological function analysis. The sample size of 8 mice per group was sufficient to provide 80% power with an α level of 0.05 to detect a mean difference of 10% in infarct volume. P < 0.05 was considered statistically significant.

## Results

**Fig. 1:**
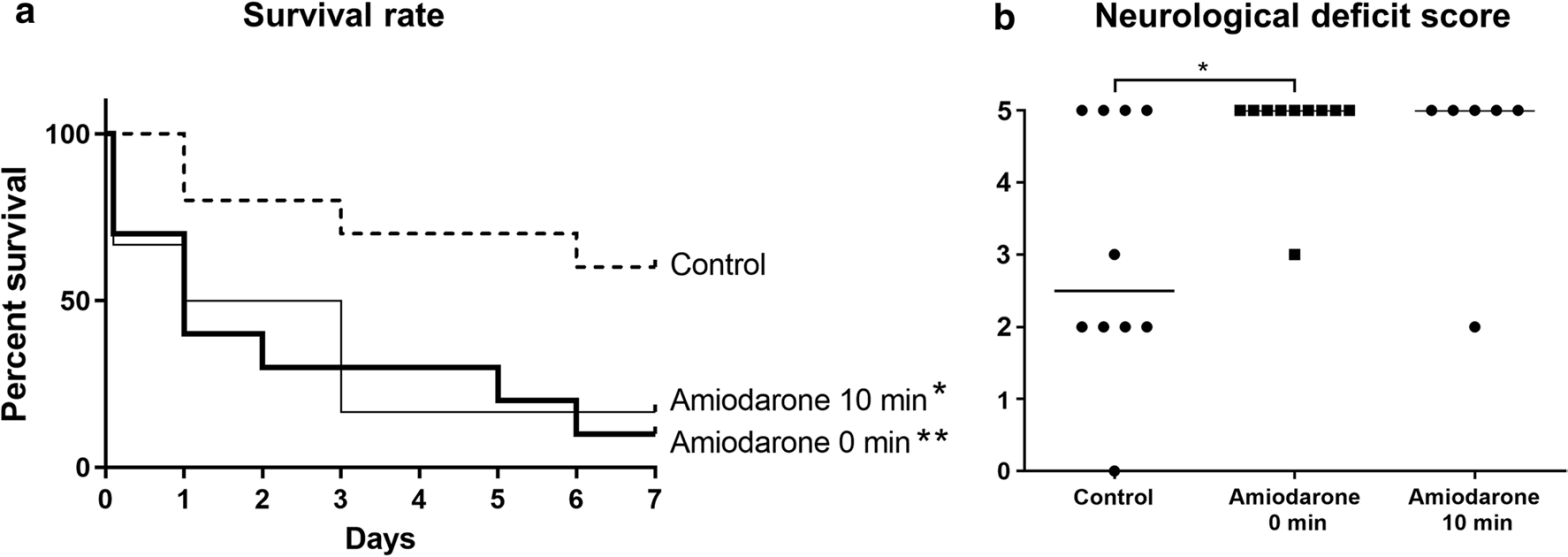
**a** Survival rate over 7 days after 40-min hypoxic–ischemic injury. **b** Neurological deficit score 7 days after 40-min hypoxic–ischemic injury. The amiodarone groups exhibited a lower survival rate over 7 days and worse neurological deficit scores (Amiodarone 0 min, Amiodarone was administered immediately after the induction of the hypoxic–ischemic injury; Amidoarone 10 min, Amiodarone was administered 10 min after the induction of the hypoxic–ischemic injury). ** P < 0.01, * P < 0.05 compared with the control group

In the 40-min hypoxic–ischemic injury model, the amiodarone groups exhibited lower survival rates over 7 days (Amiodarone 0 min: 10% (1/10), P < 0.01; Amiodarone 10 min: 17% (1/6), P < 0.05) and worse neurological deficit scores (Amiodarone 0 min: 5; 0–5, P < 0.05; Amiodarone 10 min: 5; 2–5, P = 0.142, median; range) compared with the control group (survival rate, 60% (6/10); neurological deficit score, 2.5; 1–5) (Fig. 1).

**Fig. 2:**
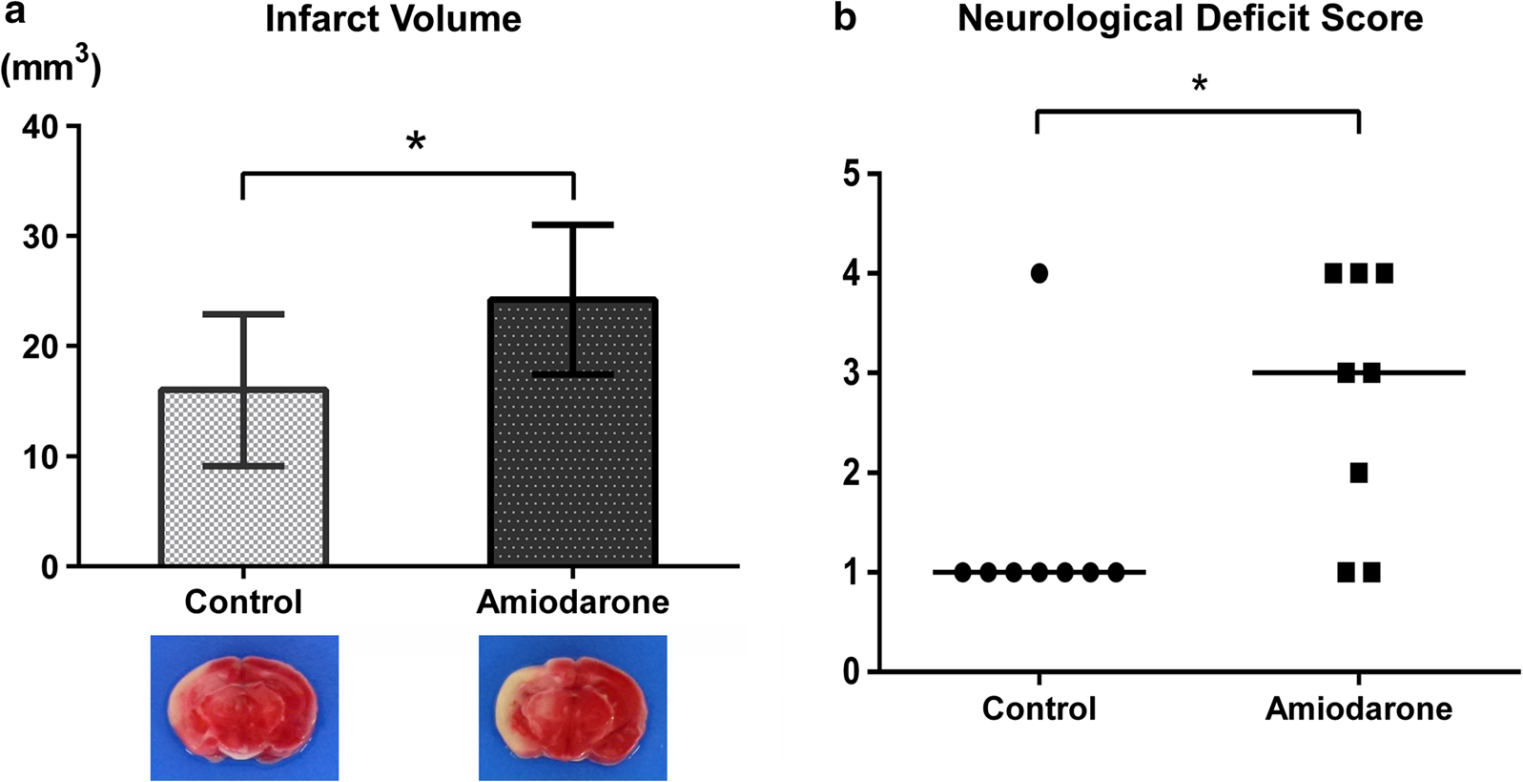
**a** Infarct volume 24 h after 25-min hypoxic–ischemic injury and representative 2,3,5-triphenyltetrazolium chloride (TTC)-stained coronal brain sections. **b** Neurological deficit score 24 h after 25-min hypoxic–ischemic injury The amiodarone group showed larger infarct volumes and worse neurological deficit scores. * P < 0.05

**Fig. 3:**
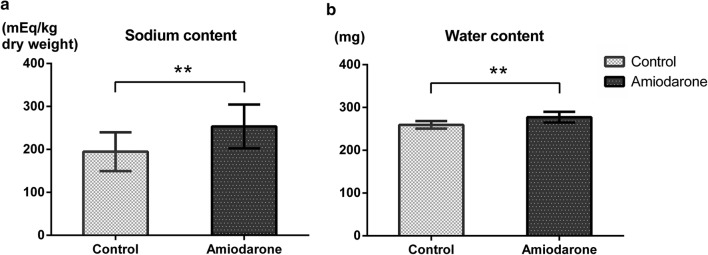
**a** Total brain sodium content 24 h after 25-min hypoxic–ischemic injury. **b** Total brain water content 24 h after 25-min hypoxic–ischemic injury The brains harvested from the amiodarone-treated mice contained larger amount of water and sodium. ** P < 0.01

As shown above, the mortality of amiodarone group mice after the 40-min hypoxic–ischemic injury was 83–90%, making it impossible to conduct further experiments to investigate the mechanism underlying the high mortality in amiodarone-treated mice. Therefore, in the next series of experiments, we induced 25-min injury and then examined infarct volume, neurological functions, and brain water/sodium content 24 h after hypoxic–ischemic injury. In the 25-min hypoxic–ischemic injury model, the amiodarone group exhibited larger infarct volumes (16.0 ± 6.9 vs. 24.2 ± 6.8 mm^3^, n = 8 each, P < 0.05) and worse neurological deficit scores (1; 1–4 vs. 3; 1–4, median; range, n = 8 each, P < 0.05) (Fig. 2). In addition, the brains harvested from the amiodarone-treated mice contained larger amount of sodium (194.7 ± 45.1 vs. 253.5 ± 50.9 mEq/kg dry weight, n = 8 each, P < 0.01) and water (259.3 ± 8.9 vs. 277.2 ± 12.5 mg, n = 8 each, P < 0.01) (Fig. 3).

There were no significant differences in baseline heart rate and mean arterial pressure between the groups (control: heart rate 510 ± 19 beats/min, mean arterial blood pressure 70 ± 6 mmHg; Amiodarone: heart rate 504 ± 43 beats/min, mean arterial blood pressure 69 ± 7 mmHg). The heart rate was significantly increased after hypoxic–ischemic insult in the control group (580 ± 43 beats/min, P < 0.05) while increase in heart rate did not reach a statistical significance in the amiodarone group (552 ± 66 beats/min, P = 0.107). The difference of the heart rates after hypoxic–ischemic between groups was not statistically significant (580 ± 43 vs. 552 ± 66 beats/min, P = 0.332). In both groups, the blood pressure increased significantly following hypoxic–ischemic insult (Control: 80 ± 6 mmHg, vs. baseline, P < 0.01; Amidoarone: 80 ± 10 mmHg, vs. baseline, P < 0.01). However, no significant difference in mean arterial pressure was observed between groups (Amidoarone vs. Control, n = 8 each, P = 0.907).

## Discussion

In the present study, we found that amiodarone administered after the onset of hypoxic–ischemic insult reduces the overall survival and worsens the brain injury and neurological outcomes.

Amiodarone suppressed the increase of heart rate induced by the hypoxic–ischemic insult. However, the heart rates after the onset of hypoxic–ischemic insult in the control and amiodarone group mice were similar, and the difference was not statistically significant. In addition, amiodarone did not affect the arterial blood pressure, indicating that the cerebral blood flow was not significantly affected by amiodarone [15]. Thus, the detrimental effects of amiodarone observed in this study were not likely through its effect on the cardiovascular function.

It has been reported that systemically administered amiodarone passes the blood–brain barrier [16, 17] and can exert its pharmacological effects on the brain [18]. In the present study, severe sodium accumulation and brain edema were associated with the higher mortality, larger infarct volumes, and worse neurological function in amiodarone-treated mice. It is likely to assume that amiodarone affected the balance of sodium ion transportation in the hypoxic–ischemic brain and led to accumulation of intracellular sodium, causing osmotic swelling and necrotic brain cell death.

The sodium pump, a transmembrane ion transporter, plays a pivotal role in balancing intracellular and extracellular sodium concentration, continuously transporting sodium out and preventing excessive accumulation of intracellular sodium [19, 20]. On the other hand, when hypoxic–ischemic brain injury occurs, the Na^+^/Ca^2+^ exchanger operates in its reverse mode, also producing sodium efflux [7]. It has been reported that the Na^+^/Ca^2+^ exchanger plays protective roles against hypoxic–ischemic brain injury [21]. Blockade of these ion transporters by amiodarone during hypoxic–ischemic brain injury can be detrimental as it may suppress the sodium efflux, causing further sodium accumulation in the brain [1, 7, 8]. Taken together, the detrimental effects of amiodarone observed in this study may be attributable to its inhibitory effects on the sodium transporters, including the sodium pump and Na^+^/Ca^2+^ exchanger.

In the present study, amiodarone administration immediately after the induction of hypoxic–ischemic insult resulted in higher mortality and neurological deficit scores compared with the administration in the later time point. The results may indicate that the blockade of sodium transporters from earlier time points would facilitate sodium accumulation and lead to severer consequences.

The amiodarone dose used in this study (50 mg/kg) was estimated to be equivalent to the dose used in cardiopulmonary resuscitation in an adult human on the body surface area basis [11]. Thus, the present results indicate that amiodarone at the clinical dose can worsen the neurological outcomes by changing the balance of sodium transportation when administered after hypoxic–ischemic insult.

On the other hand, amiodarone has multiple pharmacological effects that are considered neuroprotective. In addition to its inhibitory effects on sodium ion transporters, amiodarone blocks calcium and potassium channels. Blockade of calcium channels during hypoxic–ischemic brain injury prevents cellular hyperexcitability and ischemic glutamate release after ischemic injury [1, 22]. Recent studies also revealed beneficial effects of potassium channel blockade in the ischemic brain via microglial neuroprotection [23]. Furthermore, amiodarone possesses inhibitory effects on beta-adrenergic receptors [24], production of pro-inflammatory cytokine [25–27], and reactive oxygen species [26, 27]. These pharmacological profiles are generally associated with neuroprotection against hypoxic–ischemic brain injury rather than harmful action, and therefore not likely to be involved in the detrimental effects of amiodarone observed in the present study. The Blockade of sodium channels can also be neuroprotective. As we previously reported, pre-treatment, but not post-treatment, with amiodarone attenuates brain injury after ischemic stroke by blocking the voltage-gated sodium channel [12]. Whether amiodarone acts on the neuroprotective side or detrimental side is likely to be dependent on the clinical and pathophysiological condition. From this viewpoint, the action of amiodarone against hypoxic–ischemic brain injury should be tested from various perspectives using experimental and clinical settings.

This study has several limitations. First, as our intension for this initial study was to explore the possible effects of amiodarone at the clinical dosage against hypoxic–ischemic brain insult, we did not assess the roles of individual ion transporters and molecules that might be involved in the detrimental effect of amiodarone. Second, we used only one clinically relevant dosage (50 mg/kg). Third, we used relatively young male mice that had no degenerative vascular or cellular changes. Future expansive studies that use electrophysiological or molecular assays, different doses, aged mice, and female mice with different menopausal states may help uncover the further underlying mechanisms.

## Conclusions

Amiodarone exacerbated brain injuries and neurological outcomes after hypoxic–ischemic insults. Severe brain sodium accumulation and brain edema were associated with the detrimental effects of amiodarone. Amiodarone at the clinical dose can facilitate intracellular sodium accumulation in the brain during hypoxic–ischemic insult and exacerbate the brain injury.

## Data Availability

The datasets used and/or analyzed during the current study are available from the corresponding author on reasonable request.

## Abbreviations

ATP: adenosine triphosphate
TTC: 2,3,5-triphenyltetrazolium chloride
